# Weekends-off efavirenz-based antiretroviral therapy in HIV-infected children, adolescents, and young adults (BREATHER): a randomised, open-label, non-inferiority, phase 2/3 trial

**DOI:** 10.1016/S2352-3018(16)30054-6

**Published:** 2016-06-20

**Authors:** The BREATHER (PENTA 16) Trial Group

## Abstract

**Background:**

For HIV-1-infected young people facing lifelong antiretroviral therapy (ART), short cycle therapy with long-acting drugs offers potential for drug-free weekends, less toxicity, and better quality-of-life. We aimed to compare short cycle therapy (5 days on, 2 days off ART) versus continuous therapy (continuous ART).

**Methods:**

In this open-label, non-inferiority trial (BREATHER), eligible participants were aged 8–24 years, were stable on first-line efavirenz with two nucleoside reverse transcriptase inhibitors, and had HIV-1 RNA viral load less than 50 copies per mL for 12 months or longer. Patients were randomly assigned (1:1) to remain on continuous therapy or change to short cycle therapy according to a computer-generated randomisation list, with permuted blocks of varying size, stratified by age and African versus non-African sites; the list was prepared by the trial statistician and randomisation was done via a web service accessed by site clinician or one of the three coordinating trials units. The primary outcome was the proportion of participants with confirmed viral load 50 copies per mL or higher at any time up to the 48 week assessment, estimated with the Kaplan-Meier method. The trial was powered to exclude a non-inferiority margin of 12%. Analyses were intention to treat. The trial was registered with EudraCT, number 2009-012947-40, ISRCTN, number 97755073, and CTA, number 27505/0005/001-0001.

**Findings:**

Between April 1, 2011, and June 28, 2013, 199 participants from 11 countries worldwide were randomly assigned, 99 to the short cycle therapy and 100 to continuous therapy, and were followed up until the last patient reached 48 weeks. 105 (53%) were men, median age was 14 years (IQR 12–18), and median CD4 cell count was 735 cells per μL (IQR 576–968). Six (6%) patients assigned to the short cycle therapy versus seven (7%) assigned to continuous therapy had confirmed viral load 50 copies per mL or higher (difference −1·2%, 90% CI −7·3 to 4·9, non-inferiority shown). 13 grade 3 or 4 events occurred in the short cycle therapy group and 14 in the continuous therapy group (p=0·89). Two ART-related adverse events (one gynaecomastia and one spontaneous abortion) occurred in the short cycle therapy group compared with 14 (p=0·02) in the continuous therapy group (five lipodystrophy, two gynaecomastia, one suicidal ideation, one dizziness, one headache and syncope, one spontaneous abortion, one neutropenia, and two raised transaminases).

**Interpretation:**

Non-inferiority of maintaining virological suppression in children, adolescents, and young adults was shown for short cycle therapy versus continous therapy at 48 weeks, with similar resistance and a better safety profile. This short cycle therapy strategy is a viable option for adherent HIV-infected young people who are stable on efavirenz-based ART.

**Funding:**

UK National Institute for Health Research Health Technology Assessment; UK Medical Research Council; European Commission; PENTA Foundation; INSERM SC10-US19, France.

## Introduction

Antiretroviral therapy (ART) has substantially improved the prognosis for HIV-infected children, transforming HIV-1 infection from a life-threatening disease to a chronic infection. Furthermore, with new evidence,^1^ universal ART is now recommended^2^, ^3^ for all people living with HIV, including children and adolescents, even without major immunosuppression or HIV-related symptoms. Therefore, the population of children, adolescents, and young adults on life-long ART is growing.^4^ For this population, innovative treatment strategies are needed to address their lifestyle needs, to help maintain long-term retention-in-care, and to improve adherence to ART, which is particularly problematic during adolescence.^4^, ^5^, ^6^

Short cycle therapy aims to maintain suppression of HIV-1 RNA during planned short breaks from ART, thereby reducing ART intake, long-term toxic effects, and costs. First proof-of-concept studies suggested the feasibility of a 7 days on and 7 days off ART strategy;^7^, ^8^, ^9^ however, this approach proved inferior to continuous therapy in two randomised controlled trials in adults.^10^, ^11^ Single-arm studies with shorter breaks in ART (4 days on and 3 days off) reported inconsistent results.^12^, ^13^ However, two small randomised controlled trials confirmed that a short cycle therapy strategy of 5 days on and 2 days off ART is achievable: in the FOTO trial, including 60 US adults,^14^, ^15^ and in a larger randomised controlled trial in 103 Ugandan adults,^10^ short cycle therapy was non-inferior to continuous therapy in terms of maintained viral load suppression over 48 weeks with the added benefit of less toxicity. Most participants in both trials were on efavirenz, which has a long plasma half-life (40–91 h), and lamivudine, which has an intermediate long intracellular half-life (22 h).^16^ However, whereas participants in the US study received tenofovir disoproxil fumarate as the third drug (intracellular half-life 60–180 h),^16^ those in the Ugandan trial received shorter-acting stavudine or zidovudine.

**Research in context**

**Evidence before this study**

We searched PubMed up to March 1, 2016, with the search terms “HIV” AND (“short” AND “cycle” OR “short-cycle”) AND “therapy” and the references from the retrieved manuscripts. More than a decade ago, small proof-of-concept studies in adults suggested that structured treatment interruptions with 7 days on and 7 days off cycles of antiretroviral therapy (ART) could maintain virological suppression, particularly if drugs with long half-lives were used. However, this strategy proved inferior to continuous therapy in two randomised controlled trials in adults. Single-arm studies of a short-cycle therapy strategy with 4 days on and 3 days off showed inconsistent results: although there was no confirmed viral rebound in adults on different ART regimens in a French study, a study in highly treated adolescents and young adults on protease inhibitor-based therapy in the USA showed high rates of viral rebound. Adult studies of short cycle therapy with 2 days per week off efavirenz-based ART showed promising results: following a single arm study of 5 days on and 2 days off ART, which showed rates of virological suppression of about 90% over 48 weeks, two small randomised controlled trials in adults (one US, one Ugandan) confirmed non-inferiority of maintaining virological suppression with this short cycle therapy strategy compared with continuous therapy. No published trials have assessed 5 days on and 2 days off ART in children or adolescents.

**Added value of this study**

To our knowledge, this is the first randomised controlled trial to investigate the feasibility and acceptability of efavirenz-based short cycle therapy (5 days on and 2 days off) in a geographically diverse group of children, adolescents, and young adults with no previous treatment failure. The short cycle therapy was acceptable and non-inferior in terms of maintaining virological suppression (including to very low viral loads). No significant differences were noted in immune activation, total HIV-1 DNA, or development of resistance, and the short cycle therapy group had fewer ART-related adverse events than did the continuous therapy group. Additionally, participants expressed a strong preference for this short cycle therapy compared with continuous treatment, once they had adapted to the new routine.

**Implications of all the available evidence**

The findings of this trial, supported by previous adult studies, show that a short cycle therapy strategy with 5 days on and 2 days off efavirenz -based ART with a standard dose of efavirenz (maximum 600 mg adult equivalent daily dose) is a viable option for virologically suppressed children, adolescents, and young adults with 29% reduction in the cost of drugs. 2 year extended follow-up of the trial is ongoing to address sustainability of this strategy over a longer duration and results will be available in 2017. Further studies are warranted to assess short cycle therapy with lower doses of efavirenz and other long-acting ART regimens in settings with less frequent viral load testing than the quarterly monitoring done in trials reporting to date.

No randomised trials of short cycle therapy have been done in children or adolescents, who face longer-term ART than adults. We aimed to assess whether short cycle therapy on first-line efavirenz-based ART in children, adolescents, and young adults was non-inferior to continous therapy in terms of maintaining virological suppression and adherence to ART, while improving quality of life.

## Methods

### Study design and participants

In this open-label, randomised, parallel group non-inferiority phase 2/3 trial (BREATHER [BREaks in Adolescent and child THerapy using Efavirenz and two nRtis] PENTA 16), participants aged 8–24 years were eligible if they had a CD4 cell count 350 cells per μL or higher, suppressed viral load less than 50 copies per mL for at least 12 months on an efavirenz based regimen with two or three nucleoside or nucleotide reverse transcriptase inhibitors (NRTIs) and no previous treatment failure (first-line ART). Children on nevirapine or boosted protease inhibitor ART who had not had treatment failure and with undetectable viral load could be enrolled if they substituted efavirenz and viral load remained undetectable for 12 weeks or longer before enrolment. Previous two-drug ART, substitution of NRTIs, or both were allowed, provided these were not for regimen failure. Previous monotherapy was only allowed if taken perinatally for prevention of mother-to-child-transmission. Participants were not eligible if they were pregnant, on concomitant medications for acute illness, or if their creatinine or liver transaminases results were grade 3 or higher at screening. Parents or guardians and older participants provided written consent; young children gave assent appropriate for age and knowledge of HIV status, as per guidelines for each participating country.

The trial protocol was approved by the ethics committees in participating centres in Europe, Africa, and the Americas, and is available online.

### Randomisation and masking

Patients were randomly assigned (1:1) to remain on continuous therapy or change to short cycle therapy and randomisation was done centrally by the MRC Clinical Trials Unit at UCL (London, UK), according to a computer-generated randomisation list, using permuted blocks of varying size, stratified by age (8–12 years, 13–17 years, 18–24 years) and site (African *vs* non-African). The randomisation list was prepared by the trial statistician and securely incorporated within the database. Randomisation of study participants was done via a web service accessed by site clinician or one of the three coordinating trials units.

### Procedures

An initial 3 week randomised pilot safety phase in selected clinical centres was done in 32 participants (in which 15 participants were allocated to the short cycle therapy group) to ensure those in the short cycle therapy group maintained undetectable viral load (<50 copies per mL) after the 2 day break (Saturday and Sunday) and before resuming weekday ART on Monday. Recruitment to the main trial commenced after review of three consecutive Monday morning viral load results per participant by the Independent Data Monitoring Committee (IDMC).

In the main trial, participants randomly assigned to short cycle therapy chose 2 consecutive days off ART (Friday and Saturday or Saturday and Sunday; occasionally 2 other days: referred to as weekends off), and continued this cycle throughout. Participants on continuous therapy remained on continuous efavirenz-based ART. Substitutions for simplification (except efavirenz) or toxicity (all drugs) were allowed. Participants were randomised 2–4 weeks after screening and assessed clinically at weeks 4 and 12, then every 12 weeks until the last participant had completed 48 weeks' follow-up. Examination for lipodystrophy, Tanner stage, and a pregnancy test (in postmenarchal girls) were done at randomisation and repeated every 24 weeks.

Viral load and T lymphocytes were measured at every visit; participants with viral load of 50 copies per mL or higher had a repeat test within 1 week; those on short cycle therapy with confirmed viral rebound (two test results with viral load >50 copies per mL) recommenced continuous ART. Additional assessment of treatment adherence and a stored sample for resistance testing were requested for all participants with viral rebound. Haematology and biochemistry tests were done at screening and randomisation; thereafter, haematology was done every 12 weeks and biochemistry as per local practice. Blood lipids, including total cholesterol, high density lipoprotein, low density lipoprotein, and very low density lipoprotein, were measured at weeks 0, 24, and 48. Plasma and cells were stored for additional immunology and virology tests (see below) at baseline and weeks 4, 8, and 12, and then every 12 weeks for plasma and 24 weeks for cells. Questions on compliance to the strategy were asked at every follow-up visit. Adherence questionnaires were completed by carers and participants at weeks 0, 4, 12, 24, and 48. Acceptability questionnaires for those randomised to short cycle therapy were completed at randomisation and at final visit (or at time of change from short cycle therapy to continous therapy if earlier).

The trial incorporated three substudies. The virology and immunology substudy assessed low level viraemia (viral load <20 copies per mL), total HIV-1 DNA, and 19 biomarkers of inflammation, vascular injury, and disordered thrombogenesis; all were measured retrospectively on stored plasma and cell samples. The ultrasensitive quantitative HIV-1 RNA and DNA assays used the Qiagen QIAsymphony SP (Hilden, Germany) for nucleic acid extraction. An ABI Prism 7500 real-time thermal cycler (Foster City, CA, USA) was used for amplification of HIV-1 RNA and DNA using Invitrogen RT-PCR (Waltham, MA, USA) and Qiagen Multiplex PCR (Hilden, Germany) reagents, respectively. An in-house standard curve calibrated against the WHO HIV International standard in IU per mL was used for HIV-1 RNA quantification (appendix). The quantitation of HIV-1 DNA was based on a standard curve using the 8E5 cell line, which carries one HIV proviral genome per cell; cell numbers were estimated with the single copy gene for pyruvate dehydrogenase; results were reported as copies of HIV-1 DNA per million cells.

19 biomarkers (thrombomodulin, ICAM-1, ICAM-3, VCAM-1, CD62E, CD62P, VEGF, angiopoietin 1 and 2, serum amyloid, C-reactive protein, interleukin 1Ra, interleukin 6, interleukin 8, interleukin 10, TNFα, MCP-1, tissue factor, and D-dimers) were analysed with Meso Scale Discovery (Gaithersburg, MD, USA) or by ELISA kits (Quantikine ELISA Human Coagulation Factor III/Tissue Factor [R&D Systems, MN, USA] and TECHNOZYM D-dimer ELISA assay Technoclone [Vienna, Austria]). CD4 and CD8 lymphocyte subsets were quantified locally on fresh samples; CD45RA and CD45RO subpopulations of CD4 and CD8 cells were assessed on fresh or stored frozen cell samples at selected sites.

The adherence substudy assessed adherence in participants from selected sites by recording bottle openings using a Medication Event Monitoring System (MEMS) capped container. MEMS caps were placed on the container with most frequently taken antiretrovirals.

The longitudinal qualitative substudy focused on participants' experiences of the trial and acceptability of short cycle therapy.^17^

### Outcomes

The primary endpoint was confirmed viral load of 50 copies per mL or higher by week 48. Secondary outcomes were: confirmed viral load of 400 copies per mL or higher by week 48; cumulative number and type of major HIV-1 RNA resistance mutations in those with viral rebound; change in CD4% and CD4 cell count, glucose, blood lipids from baseline to week 48; changes in ART regimen; change back to continuous therapy (short cycle therapy only); adherence; acceptability (short cycle therapy); division of AIDS grade 3 or 4 clinical or laboratory adverse events,^18^ and treatment-modifying adverse events of any grade; and new US Centers for Disease Control (CDC) stage B or C diagnoses or death.

### Statistical analysis

160 participants (80 per group) provided 80% power to exclude a non-inferiority margin of 12% for the difference in proportion of participants reaching the primary endpoint, assuming 10% of participants have confirmed viral load 50 copies per mL or higher in the continuous therapy group and a one-sided α of 0·05 (two-sided α=0·1). The Trial Steering Committee decided to continue recruitment until the end of the planned randomisation period to allow sites to recruit patients already invited for screening and to avoid the study being underpowered if the proportion of participants reaching the primary endpoint in the continuous therapy group was lower than expected (the Trial Steering Committee did not have access to event rates during the trial).

In the primary, intent-to-treat analysis, the proportion of participants who had viral rebound (≥50 copies per mL) was estimated with Kaplan-Meier methods, with adjustment for baseline stratification factors, censoring at week 54 (upper band of week 48 assessment window) or last follow-up date if not seen at week 48. The difference in proportion (between the short cycle therapy group and continuous therapy group) of participants who had viral rebound was estimated and two-sided 90% CIs of the difference was obtained with bootstrap SE (1000 replicates).^19^ In a prespecified sensitivity analysis on the per-protocol population, individuals were censored if they had a break in treatment for longer than 7 days, discontinued efavirenz for longer than 7 days, or changed strategy to continuous therapy for reasons other than viral rebound. The intent-to-treat analysis was also repeated without adjustment for stratification factors. Confirmed viral load of 400 copies per mL or higher was estimated by the same approach. Major resistance mutations were summarised.

Immunology (including substudy biomarkers), HIV-DNA, haematology, biochemistry, and lipids were assessed at week 48 by fitting normal regression models with adjustment for randomised group and baseline values. Natural log transformations were applied as appropriate (for HIV-DNA, biomarkers and ratios of CD45RA [naive]:CD45RO [memory] cells and CD8RA [naive]:CD8RO [memory] cells). Change from baseline is presented as change from mean at baseline in all participants.

Categorical variables were compared with Fisher's exact tests, or McNemar's tests for paired data; rates used Poisson regression (including a random effect for participant where appropriate). Generalised estimating equations (independent correlation structure) were used to compare self-reported adherence across randomised groups over time. Stata version 13.1 was used for all analyses (StataCorp 2013, College Station, TX, USA: StataCorp LP). To assess adherence to allocated strategy, the number of days that MEMS cap was opened at least once divided by number of days that MEMS cap was in use during the trial was calculated for each day of the week.

Pilot phase data were included in the analysis. The IDMC reviewed full interim data on three occasions, viral load and enrolment data at a fourth meeting, and analyses of viral load results alone on six further occasions during the trial.

The trial was registered with EudraCT, number 2009-012947-40), ISRCTN, number 97755073, and CTA, number 27505/0005/001-0001.

### Role of the funding source

The funders had no direct role in the study design, data collection, data analysis, data interpretation, report writing, or decision to submit the report for publication. The corresponding author had access to all data and responsibility for submission for publication.

## Results

**Figure 1:**
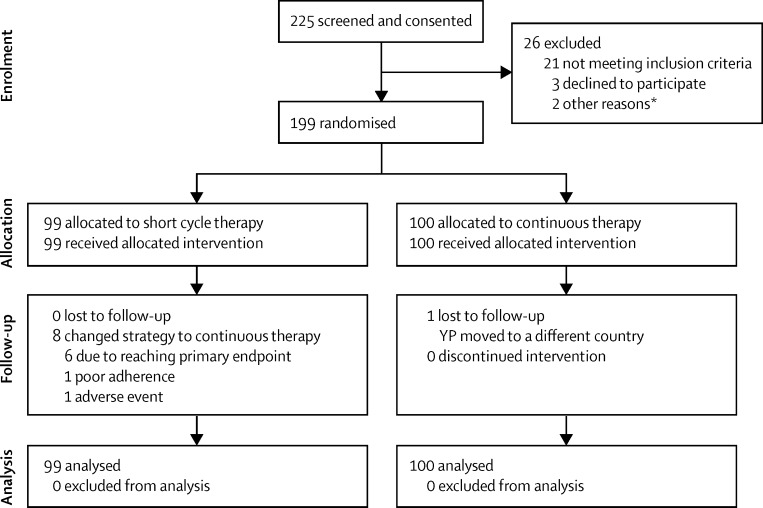
Trial profile *One participant was unable to attend the randomisation visit due to a traffic accident and another participant was excluded because of unreliable attendance.

Between April 1, 2011, and June 28, 2013, 227 participants were screened (figure 1), of whom 199 from 24 sites were randomly assigned (99 to short cycle therapy and 100 to continuous therapy). One participant in the continuous therapy group moved location and withdrew consent at week 24; the remaining 198 were followed up to at least week 48.

Of those patients randomly assigned, 70 (35%) were recruited from Uganda (35 in the short cycle therapy group and 35 in the continuous therapy group), 48 (24%) from western Europe, 36 (18%) from Thailand, 20 (10%) from Ukraine, 14 (13%) from the USA, and 11 (6%) from Argentina (appendix p 5). Baseline characteristics were similar between the groups (table 1). Although CD4% and count were high and well matched between groups, fewer participants had CDC stage C disease in the short cycle therapy group than in the continuous therapy group. Pre-trial ART exposure was comparable between groups: median time on ART at randomisation was 6·1 years (IQR 3·8–8·4), 82 (41%) were on their initial ART regimen at baseline, 29 (15%) had previously substituted a protease inhibitor, but following the exclusion criteria, none had switched ART for failure.

**Figure 2:**
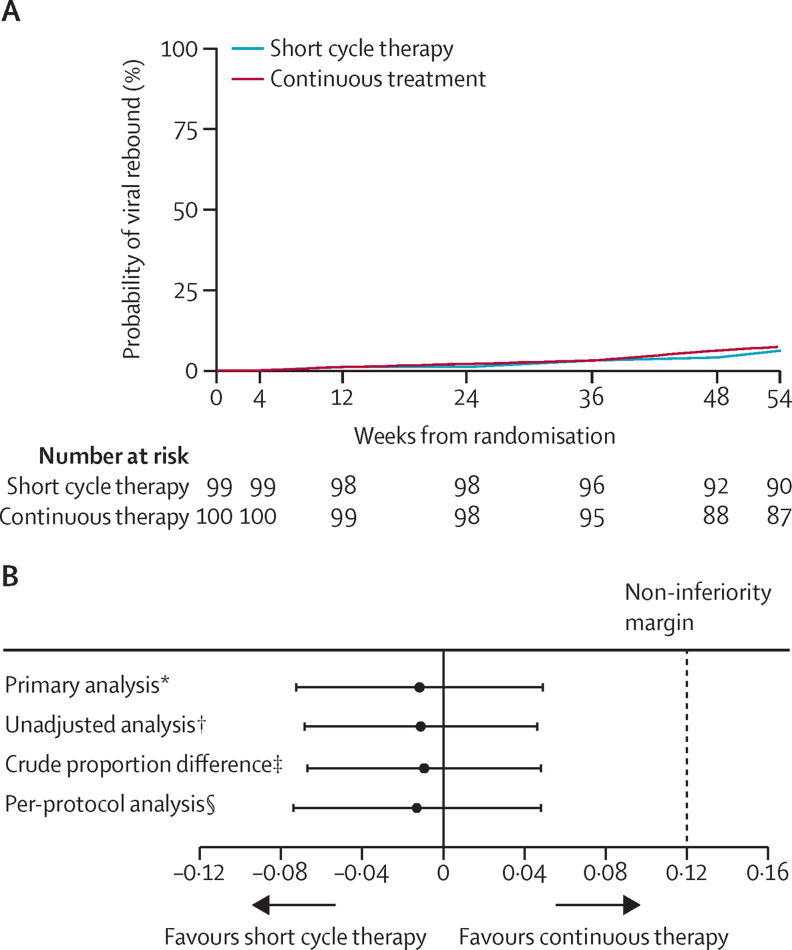
Time to viral rebound (A) Kaplan-Meier graph adjusted for stratification factors—time from randomisation to viral rebound (confirmed viral load ≥50 copies per mL). (B) Estimated difference in proportion of participants with viral rebound (two-sided 90% CI) between short cycle therapy and continuous therapy at week 48 for different analyses. *Difference in estimated probability of viral rebound, Kaplan-Meier methods, with adjustment for study stratification factors. †Difference in estimated probability of viral rebound, Kaplan-Meier methods. ‡With exact confidence intervals. §Kaplan-Meier methods, censoring individuals who violated the profile at that time, with adjustment for study stratification factors.

13 participants had a confirmed viral load 50 of copies per mL or higher at any time up to 48 weeks (table 2), an estimated probability of viral rebound of 6·1% in short cycle therapy versus 7·3% in continuous therapy (difference −1·2%, 90% CI −7·3 to 4·9, test for difference, bootstrap p=0·75; figure 2A). Thus, the 4·9% upper band of the two-sided 90% confidence limit was well within the 12% non-inferiority margin. The per-protocol analysis gave a similar estimated difference of −1·1% (90% CI −6·8 to 4·6), as did analysis without adjustment for stratification factors (figure 2B).

After viral rebound, five (83%) of six participants in the short cycle therapy group resuppressed viral load (three on the same regimen but changed to continuous daily ART, two following regimen change) compared with only three (43%) of seven participants in the continuous therapy group (two resuppressed while continuing the same regimen and one after regimen change). The remaining five participants (one in the short cycle therapy group and four in the continuous therapy group) remained non-suppressed; three on first-line ART (one in the short cycle therapy group and two in the continuous therapy group) and two (in the continuous therapy group) after switching to second-line ART. Results repeating the primary analysis, adjusted for CDC stage at baseline were qualitatively unchanged: −1·3% difference between groups, in favour of short cycle therapy (90% CI −7·4 to 4·7, test for difference, bootstrap p=0·72).

To determine whether the risk of reaching the primary endpoint was related to type of NRTI (short-acting or long-acting), a Cox model adjusted for randomised group and NRTI received (zidovudine *vs* abacavir or tenofovir disoproxil fumarate) was fitted (exploratory analysis); results showed no significant differences between continuous therapy and short cycle therapy (p=0·81; data not shown).

Six participants (two [2%] in the short cycle therapy group and four [4%] in the continuous therapy group) had confirmed viral load of 400 copies per mL or higher by week 48; estimated probability 2·1% in the short cycle therapy group versus 4·2% in the continuous therapy group (difference −2·1%, 90% CI −6·2 to 1·9, p=0·38).

12 participants changed ART regimen during the first 48 weeks (three in the short cycle therapy group and nine in the continuous therapy group, Fisher's exact p=0·13), five because of toxic effects (one in the short cycle therapy group and four in the continuous therapy group; Table 2, Table 3).

Of 13 participants reaching the primary endpoint, resistance results were available for nine (three in the short cycle therapy group and six in the continuous therapy group); the remaining four patients had samples with low viral load, insufficient to obtain a result (three in the short cycle therapy group: 56 copies per mL, 62 copies per mL, and 126 copies per mL; one in the continuous therapy group: 231 copies per mL). All four participants suppressed again after these blips, suggesting drug resistance was unlikely. Seven of nine participants with available results had resistance mutations: all seven had NNRTI mutations and two had Met184Val (table 2).

No new CDC stage C and two CDC stage B events were recorded (bronchopneumonia in the short cycle therapy group and bronchitis in the continuous therapy group) and no significant differences were noted between groups in CD4% or CD4 cell count (table 2). With the exception of lower mean corpuscular volume in those on zidovudine and lower platelet levels in the short cycle therapy group compared with the continuous therapy group; haematological variables did not differ (appendix p 6). Concentration of low density lipoproteins was higher at week 24 in the short cycle therapy group than in the continuous therapy group, but we observed no difference at week 48 (appendix p 6).

By week 48, eight participants in the short cycle therapy group had reverted to continuous therapy: six participants reached the primary endpoint, one developed gynaecomastia leading to efavirenz discontinuation and resumption of daily ART, and one had ART changed for poor adherence (without reaching the primary endpoint).

By 48 weeks, 20 participants had 27 grade 3 or 4 adverse events, with decreased neutrophil count being the most common (two participants in the short cycle therapy group *vs* six participants in the continuous therapy group; Fisher's exact p=0·28; table 3). Two ART-related adverse events were reported in two participants in the short cycle therapy group compared with 14 events in ten participants in the continuous therapy group (Poisson p=0·02 for number of events; Fisher's exact p=0·03 for number of participants); this was the only significant difference in adverse events between groups). Lipodystrophy and gynaecomastia were the most common ART-related events. 13 serious adverse events were reported in nine participants (six in the short cycle therapy group and three in the continuous therapy group; table 3). There were five pregnancies (one in the short cycle therapy group and four in the continuous therapy group).

Among 192 children (98 in the short cycle therapy group and 94 in the continuous therapy group) in the immunology and virology substudy, values for viral load less than 20 copies per mL, total HIV-1 DNA, and inflammatory markers, including interleukin 6 and D-dimer, were similar between randomised groups at baseline (table 1; appendix p 7). At week 48, 13 (13%) children in the short cycle therapy group and 14 (15%) in the continuous therapy group had viral load 20 copies per mL or higher (Fisher's exact p=0·84) and there were no significant differences between groups in total HIV-1 DNA (table 2; p=0·13), including after adjustment for differences at baseline or after exclusion of participants with evidence of viral rebound (data not shown). No differences between groups were noted at week 48 in the 19 biomarkers of inflammation, vascular injury, and disordered thrombogenesis, with the exception of D-dimer, which was lower in the short cycle therapy group than in the continuous therapy group by log 0·5 (p=0·05; table 2; appendix p 7). No differences were identified in CD8 cells, ratios of CD45RA (naive):CD45RO (memory) cells, and CD8RA (naive):CD8RO (memory) cells between groups at week 48 (data not shown).

**Figure 3:**
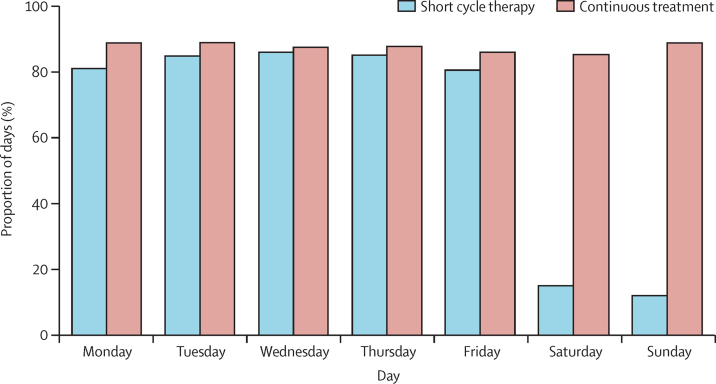
Proportion of days MEMS caps were opened Data for 31 participants in the short cycle therapy group and 30 participants in the continuous treatment group (including 23 in each group with data to 48 weeks). MEMS=Medication Event Monitoring System.

In the short cycle therapy group, 95% of weekend breaks were reported as taken (99% excluding time after return to continuous therapy). The MEMS cap substudy data supported these results. Among 61 participants enrolled in the substudy (31 in the short cycle therapy group and 30 in the continuous therapy group), 56 (28 in each group) continued to use MEMS caps until 36 weeks and 46 (23 in each group) were still using MEMS caps at week 48. The median number of cap openings per week was five (IQR 4–5) in the short cycle therapy group and seven (6–7) in the continuous therapy group. MEMS caps were opened at least once daily from Monday to Friday more than 80% of the time in both groups, with the percentage of bottle openings remaining high in the continuous therapy group at weekends, but dropping to less than 20% for those on short cycle therapy (figure 3).

Based on ART logs, updated at each visit, one participant in the short cycle therapy group and seven participants in the continuous therapy group had a treatment interruption of 3 days or more (excluding weekend breaks in the short cycle therapy group; Fisher's exact test p=0·07). Adherence questionnaires were completed by 91 participants in the short cycle therapy group and 93 participants in the continuous therapy group at one or more visit (80 in both groups at four or more visits) to 48 weeks. Adherence was similar in both groups with 7% (29 of 414) of reports in the short cycle therapy group versus 10% of (40 of 409) reports in the continuous therapy group of missing ART in the week prior to the assessment visit (excluding weekend breaks in the short cycle therapy group; p=0·42). Adherence based on carers' questionnaires was also similar between the two groups (data not shown).

In acceptability questionnaires completed at baseline, 70 (88%) of 80 participants in the short cycle therapy group thought the approach would be easier than staying on continuous therapy. At end of follow-up 81 (90%) of 90 participants in the short cycle therapy group reported that weekend breaks made life easier than daily ART, mainly because going out with friends was easier: 15 (20%) of 76 participants who completed both questionnaires reported this was difficult pre-trial compared with only two of 76 during the trial (McNemar p=0·001; appendix p 8). The acceptability of short cycle therapy as further explored in the qualitative substudy will be reported elsewhere.^17^

## Discussion

We found no evidence that short cycle therapy was inferior to continuous therapy in maintaining viral load suppression with a very small non-significant difference between the groups favouring short cycle therapy. Further, five of six participants on short cycle therapy who had low level viraemia resuppressed on returning to daily ART. Results were essentially unchanged in further analyses that adjusted for small differences in CDC stage at baseline, and were done per protocol. Our results have broad generalisability because we recruited participants from diverse geographical, ethnic, and sociocultural backgrounds in 11 countries, including 21% who were young adults in their early twenties.

There were fewer major resistance mutations among children failing on short cycle therapy than in those on continuous therapy, although this was not statistically significant. In both groups and similarly to the PENPACT-1 trial, which assessed timing of switch to second-line ART, NNRTI and Met184Val mutations emerged rapidly even at low level viraemia.^20^

Although virological suppression to less than 50 copies per mL was the primary endpoint, we further investigated the safety of short cycle therapy by assessing its effect on very low level viraemia and HIV reservoir and showed no differences between the short cycle therapy and continuous therapy groups. Methods of varying technical difficulty and biological meaning have been suggested to quantify the HIV reservoir, which is responsible for viral rebound following treatment interruption.^21^ We measured HIV-1 DNA because it is a surrogate for reservoir size in acute and chronic HIV infection.^22^, ^23^

Increases in chronic immune activation and inflammation have been reported in adult interruption trials designed to allow rebounds in viral load, and have been associated with adverse HIV-related outcomes.^24^ Immune activation with raised concentrations of biomarkers of inflammation and coagulation has also been reported in patients with virological suppression,^25^ both among elite controllers not on ART and ART recipients with supressed viral load, albeit at low levels.^26^ Therefore, we also measured the effect of the short cycle therapy strategy on 19 potentially important biomarkers and found no evidence of any differences between groups, with the exception of D-dimer which, by contrast with expectation, was lower in short cycle therapy than in continuous therapy, which could be a chance finding. The absence of a signal suggestive of any increased immune activation and inflammation adds further confidence that the short cycle therapy strategy was not causing subclinical injury. Furthermore, we recorded no differences in cellular markers previously shown to be rapidly deranged during treatment interruption.^27^

Most safety profiles were similar between randomised groups, and there were more ART-related adverse events reported in the continuous therapy group. However, in an open-label trial, potential for reporting bias exists.

Assuring adherence to the randomised strategy is crucial to the integrity of trial results. If participants randomly assigned to the continuous therapy group elected, of their own accord, to take breaks in therapy, non-inferiority of short cycle therapy and continuous therapy might be shown, because both groups could be taking similar breaks off-ART. Three independent indicators of adherence to assigned strategy (self-reported adherence, MEMS caps substudy, and differences between groups in mean corpuscular volume among zidovudine recipients) all showed that participants on short cycle therapy had appropriately less ART exposure than those on continuous therapy.

As well as being the first randomised trial in children, our results build on those from two adult trials with similar design, showing non-inferiority of short cycle therapy versus continuous therapy on efavirenz-based ART.^10^, ^15^ Only one non-randomised study of short cycle therapy in US adolescents and young adults has been reported in heavily ART-experienced participants taking a 3 day weekend break from protease inhibitor-based ART regimens.^12^ This study differed substantially from our study and the adult short cycle therapy trials in both design and ART used. More than a third of participants had viral rebound and more than half changed to continuous treatment for other reasons; with no control group and multiple previous ART regimens, viral load, and resistance test results are hard to interpret or compare with our trial. Protease inhibitor ART might not be ideal for short cycle therapy because half-lives are shorter than NNRTIs and might not protect against viral replication during days off. Furthermore, participants in the US study had breaks of 3 days, whereas those in our trial had breaks of only 2 days.

Acceptability of the short cycle therapy strategy was shown among participants from all backgrounds; in particular, it was valued because it allowed for more socialising with friends at weekends. Similar results were reported in the associated qualitative substudy, during which participants also discussed liking short cycle therapy because of perceived reduction of previously unreported and unrecognised ART side-effects, such as dizziness and reduced energy. The qualitative substudy^17^ provided insights into the complexities of physician–patient interactions, particularly relating to non-adherence. In particular, participants who are virologically suppressed might elect not to disclose adherence lapses because of a desire not to fail or disappoint their physician. Overall, the qualitative findings endorsed participants' enthusiasm for short cycle therapy, but also highlighted the need for support with early adaptation to weekend breaks off-ART.^17^

The overall reduction in drug exposure could reduce long-term toxicity for individuals and, at a population level, result in cost savings, enabling more participants to receive treatment. The ENCORE1 trial^28^ showed that daily 400 mg efavirenz was non-inferior to 600 mg, with less toxicity; in both groups, efavirenz was given with daily tenofovir and emtricitabine. Efavirenz 400 mg daily is included as an alternative option to 600 mg efavirenz-based ART in revised WHO 2016 adult guidelines.^29^ Of note, the weekly cumulative dose of daily 400 mg efavirenz is almost the same as in our trial. Both strategies seem to be more acceptable to patients than 600 mg daily efavirenz and provide the possibility of individualisation of ART regimens to suit life situations.

The results from this study show that short cycle therapy might be a promising strategy for adherent children and adolescents well established on ART. However, follow-up is relatively short. A 2-year trial extension is ongoing, which will provide further data on longer-term sustainability. More than 90% of participants have reconsented to stay on their randomised strategy and we expect results in 2017. Of note, this short cycle therapy strategy can be generalised only to children and young people taking efavirenz-based ART who have not had treatment failure, and where there is availability of viral load monitoring. Appropriate counselling and support is needed to explain that there should be a maximum of 2 days per week breaks in therapy. Furthermore, results presented here cannot necessarily be extrapolated to ART containing the reduced dose of efavirenz (equivalent to 400 mg for adults) or other ART regimens, or to settings where viral load monitoring is unavailable or infrequent. Further research is needed to address this, and could also assess short cycle therapy with other suitable long-acting drugs or drugs with a higher barrier to resistance such as tenofovir alafenamide and dolutegravir .^30^

In conclusion, in an adherent and geographically diverse population of HIV-infected 8–24 year-olds on 600 mg efavirenz-based ART, a short cycle therapy strategy with 2-day weekend breaks was non-inferior to continuous therapy in terms of virological, immunological, inflammatory effects, and resulted in fewer adverse events. Treatment with ART 5 days per week instead of 7 provides potential for cost savings. Short cycle therapy was liked by participants; in particular, it improved their social lives. This short cycle therapy strategy is a viable option for adherent HIV-infected young people who are stable on efavirenz-based ART. Ongoing longer-term follow-up will further inform sustainability and further research is required for settings where viral load monitoring is less accessible.

Correspondence to: Dr Anna Turkova, MRC Clinical Trials Unit at UCL, London, WC2B 6NH, UK **a.turkova@ucl.ac.uk**

## Figures and Tables

**Table 1:** Baseline characteristics

		**Short cycle therapy (n=99)**	**Continuous therapy (n=100)**	**Total (n=199)**
Male		57 (58%)	48 (48%)	105 (53%)
Age (years)		13·7 (11·7–17·7)	14·4 (12·0–17·5)	14·1 (11·9–17·6)
	8–12	38 (38%)	39 (39%)	77 (39%)
	13–17	39 (39%)	41 (41%)	80 (40%)
	18–24	22 (22%)	20 (20%)	42 (21%)
Ethnic origin				
	Black (African or other)	58 (59%)	54 (54%)	112 (56%)
	White	24 (24%)	17 (17%)	41 (21%)
	Asian	15 (15%)	22 (22%)	37 (19%)
	Other	2 (2%)	7 (7%)	9 (5%)
Route of infection				
	Vertical	90 (91%)	90 (90%)	180 (90%)
	Sexual contact	7 (7%)	7 (7%)	14 (7%)
	Unknown/other*	2 (2%)	3 (3%)	5 (3%)
CDC stage†				
	N	16 (16%)	10 (10%)	26 (13%)
	A	25 (25%)	25 (25%)	50 (25%)
	B	45 (45%)	43 (43%)	88 (44%)
	C	13 (13%)	21 (21%)	34 (17%)
Cumulative ART exposure before baseline (years)		6·2 (3·8–7·9)	5·9 (4·0–8·4)	6·1 (3·8–8·4)
Baseline regimen is the initial ART regimen		40 (40%)	42 (42%)	82 (41%)
Efavirenz plus				
	Zidovudine plus lamivudine	52 (53%)	53 (53%)	105 (53%)
	Tenofovir plus lamivudine or emtricitabine	25 (25%)	27 (27%)	52 (26%)
	Abacavir plus lamivudine or emticitabine	22 (22%)	18 (18%)	40 (20%)
	Other‡	0 (0%)	2 (2%)	2 (1%)
CD4 percentage		34·5 (29·3–39·0)	34·0 (29·5–38·1)	34·0 (29·5–38·5)
	<25%	5 (5%)	6 (6%)	11 (6%)
	≥25% to <40%	73 (74%)	76 (76%)	149 (75%)
	≥40%	21 (21%)	18 (18%)	39 (20%)
CD4 cell count (cells per μL)		722·5 (581·0–965·0)	747·3 (575·3–972·8)	735·0 (575·5–967·5)
	≥350–500	16 (16%)	12 (12%)	28 (14%)
	>500	83 (84%)	88 (88%)	171 (86%)
Viral load (copies per mL)				
	<20§	91 (93%)	86 (91%)	177 (92%)
	≥20	7 (7%)	8 (9%)	15 (8%)
Total HIV-1 DNA (copies per million cells)		420 (159–871)	309 (136–926)	347 (145–894)
Interleukin 6 (pg/mL)		0·6 (0·4–0·9)	0·6 (0·4–0·9)	0·6 (0·4–0·9)
D-dimers (ng/mL)		69·1 (3·13–135·4)	65·7 (4·8–80·3)	67·5 (3·1–152·2)
CRP (pg/mL)		631·2 (303·8–2676·1)	621·6 (260·8–2164·1)	626·8 (288·8–2311·0)

Data are median (IQR) or n (%). CDC=US Centers for Disease Control and Prevention. ART= antiretroviral therapy. CRP=C-reactive protein.

* Three young people acquired HIV through blood products (one in the short cycle therapy group and two in the continuous therapy group), two had uncertain methods of transmission (one in the continuous therapy group and one in the short cycle therapy group).

† One young person in the continuous therapy group with unknown US CDC stage at randomisation.

‡ The remaining nucleotide reverse transcriptase inhibitor backbones in two patients were (1) zidovudine plus lamivudine plus tenofovir and (2) didanosine plus abacavir.

§ Seven samples (one in the short cycle therapy group and six in the continuous therapy group) were not available for testing with an ultra-sensitive assay.

**Table 2:** Trial efficacy from randomisation to week 48 assessment

				**Short cycle therapy (n=99)**	**Continuous therapy (n=100)**	**p value**
Primary endpoint						
	Participants with confirmed viral load ≥50 copies/mL			6 (6%)	7 (7%)	0·75
Secondary endpoints						
	Participants with confirmed viral load ≥400 copies/mL			2 (2%)	4 (4%)	0·38
	Participants with change in ART regimen			3 (3%)	9 (9%)	0·13
		Viral rebound		0	1	..
		Toxicity*		1	4	..
		Adherence problems		1	1	..
		Simplification		1	3	..
	Participants with mutations present at viral rebound† (participants with resistance test result available)			2 (3)	5 (6)	1·00
		Number of NNRTI mutations				
			None	1	1	..
			1–2	1	5	..
			3 or more	1	0	..
		Number of NRTI mutations				
			None	2	5	..
			1	1	1	..
	Mean change in CD4 percentage (%)			0·2% (0·4)	0·1% (0·4)	0·76
	Mean change in absolute CD4 count (cells per μL)			−34·2 (20·9)	−21·6 (21·1)	0·67
Substudy results				n=98	n=94	
	Viral load					
		≥20 copies/mL at week 48		13 (13%)	14 (15%)	0·84
		<20 copies/mL at week 48		85 (87%)	80 (85%)	
	Mean change in total HIV-1 DNA (Ln copies per million cells)			0·1 (0·1)	−0·2 (0·1)	0·13
	Mean change in interleukin 6 (Ln pg/mL)			0·0 (0·1)	0·1 (0·1)	0·64
	Mean change in D-dimers (Ln ng/mL)			−0·5 (0·2)	−0·0 (0·2)	0·05

Data are n (%) or mean change from randomisation (SE), unless otherwise stated. ART=antiretroviral therapy. NRTI=nucleoside/nucleotide reverse transcriptase inhibitors. NNRTI=non-nucleoside reverse transcriptase inhibitor. Ln=natural logarithm.

* One gynaecomastia in the short cycle therapy group, three lipodystrophy events with onset before baseline in the continuous therapy group, and one raised transaminases in the continuous therapy group.

† Two participants on short cycle therapy: (1) Leu100Ile, Lys103Asn, Tyr188Cys and (2) Lys103Asn, Met184Val; five participants on continuous therapy: (1) Val106, Glu138Ala, (2) Lys103Asn, Val106Met, (3) Met230Leu, (4) Lys103Asn, Val106Met, and (5) Met184Val, Glys190Ser; samples from four additional patients (three in the short cycle therapy group and one in the continuous therapy group) with low level viraemia failed to amplify.

**Table 3:** Adverse events from randomisation to week 48 assessment

		**Short cycle therapy (n=99)**	**Continuous therapy (n=100)**	**Total (n=199)**
Grade 3 and 4 adverse events		13 (8)	14 (12)	27 (20)
Clinical				
	Infections and infestations	3	1	4
	Nervous system disorders*	2	1	3
	Skin and subcutaneous tissue disorders	0	1	1
	Surgical and medical procedures	0	2	2
	Kaposi's sarcoma (AIDS related)	1	0	1
	Suicidal ideation	0	1	1
	Gynaecomastia	1	0	1
Laboratory				
	Neutropenia	2	6	8
	Low density lipoprotein cholesterol increased	1	1	2
	Bilirubin increased	1	0	1
	Calcium decreased	1	0	1
	Glucose decreased	1	0	1
	Alkaline phosphatase increased	0	1	1
ART-related adverse events (all grades)		2 (2)	14 (10)	16 (12)
	Lipodystrophy†	0	5	5
	Gynaecomastia	1	2	3
	Suicidal ideation	0	1	1
	Dizziness	0	1	1
	Headache and syncope	0	1	1
	Spontaneous abortion	1	1	2
	Neutropenia	0	1	1
	Raised transaminases	0	2	2
Treatment-modifying adverse events (all grades)‡		1 (1)	4 (4)	5 (5)
Serious adverse events§		7 (6)	6 (3)	13 (9)
Serious adverse event rate per 100 person-years (95% CI)		6·9 (3·3–14·4)	5·9 (2·6–13·1)	6·4 (3·7–10·9)

Data are number of episodes (number of participants), unless otherwise stated. ART=antiretroviral therapy. The only significant difference in numbers of adverse events or number of patients with adverse events were in ART-related adverse events (Poisson p=0·02, Fisher's exact p=0·03, respectively).

* One headache (short cycle therapy group), one hemiparesis (short cycle therapy group), and one collapse or suspected seizure (continuous therapy group).

† Lipodystrophy events (continuous therapy group): two new and three worsening events with onset before baseline.

‡ One gynaecomastia (short cycle therapy group), three worsening lipodystrophy events with onset before baseline (continuous therapy group), and one raised concentration of transaminases (continuous therapy group).

§ Serious adverse events in the short cycle therapy group: five admissions to hospital (one headache, one exacerbation of bronchiectasis, one Kaposi's sarcoma, one measles, and one epistaxis) and two other important medical conditions (one spontaneous abortion and one transient hemiparesis); in the continuous therapy group: one life threatening (suicidal ideation), four admissions to hospital (one contusion of chest, one collapse or suspected seizure, one spontaneous abortion, and one appendicitis) and one other important medical condition (neurosyphilis).
