# Supplementary material for: Weekends-off efavirenz-based antiretroviral therapy in HIV-infected children, adolescents, and young adults (BREATHER): a randomised, open-label, non-inferiority, phase 2/3 trial
Source: Lancet HIV. 2016 Jun 20;3(9):e421–30. doi: 10.1016/S2352-3018(16)30054-6 (PMC4995440; doi:10.1016/S2352-3018(16)30054-6)
Supplement: Supplementary appendix [file mmc1.pdf]

THE LANCET HIV

Supplementary appendix

This appendix formed part of the original submission and has been peer reviewed. We post it as supplied by the authors.

Supplement to: The BREATHER (PENTA 16) Trial Group. Weekends-off efavirenz-based antiretroviral therapy in HIV-infected children, adolescents, and young adults (BREATHER): a randomised, open-label, non-inferiority, phase 2/3 trial. *Lancet HIV* 2015; published online June 20. http://dx.doi.org/10.1016/S2352-3018(16)30054-6.

Supplementary Appendix

CONTENT

1. Study Contributors

2. Ultrasensitive quantitative HIV-1 virological assays: additional information on methods

3. Supplementary Table 1: Centres participating in the BREATHER trial

4. Supplementary Table 2: Mean changes in MCV (fL), platelets (x109/L), and LDL cholesterol (mg/dL) from randomisation to week 48 assessment by use of zidovudine at baseline

5. Supplementary Table 3: Results from biomarkers substudy

6. Supplementary Table 4: Comparison of adherence difficulties between baseline and end of study in short-cycle therapy group.

Study Contributors

BREATHER Trial Management Group: J Ananworanich, A Babiker, S Bernays, T Bunupuradah, K Butler (chair), J Calvert, S Chalermpantmetagul, K Chokephaibulkit, R Choudhury, A Compagnucci, TR Cressey, C Giaquinto, D Gibb, L Harper, J Inshaw, J Kenny, H Kizito, N Klein, E Menson, S Montero, V Musiime, A Nanduudu, E Nastouli, M Ndiaye, JT Ramos Amador, T Rhodes, Y Riault, Y Saidi, J Seeley, K Scott, S Storey, A Turkova.
BREATHER Steering Committee: I Weller (chair), JT Ramos, J Ananworanich, K Butler, P Clayden, J Darbyshire, V Leroy, V Musiime, DM Gibb.
PENTA Steering Committee: J-P Aboulker, J Ananworanich, A Babiker, E Belfrage, S Bernardi , R Bologna, D Burger, K Butler, G Castelli-Gattinara , P Clayden, A Compagnucci, TR Cressey, R de Groot, M Della Negra, A De Rossi, A Di Biagio, D Duiculescu (deceased), A Faye, V Giacomet, C Giaquinto (chairperson), DM Gibb, I Grosch-Wörner, M Hainault, L Harper, N Klein, M Lallemant, H Lyall, M Mardarescu, L Marques, MJ Mellado Peña, M Marczynska, D Nadal, E Nastouli, L Naver, T Niehues, D Pillay, J Popieska , JT Ramos Amador, P Rojo Conejo, L Rosado, V Rosenfeldt (deceased), C Rudin, Y Saïdi, M Sharland, HJ Scherpbier, C Thorne, G Tudor-Williams, A Turkova, N Valerius, A Volokha, AS Walker, S Welch.
Independent Data Monitoring Committee: A Pozniak (Chair), S Vella, G Chène, T Vesikari.
Trials Units: INSERM SC10-US19, France: JP Aboulker, A.Arulananthan, A Compagnucci, S Léonardo, L.Meyer, M.Ndiaye, Y Riault, Y Saïdi.
MRC Clinical Trials Unit, UK: A Babiker, J Calvert, R Choudhury, D Ford, DM Gibb, L Harper, J Inshaw, D Johnson, J Kenny, S Martins, S Montero, C O'Leary, K Scott, J Thompson, A Turkova, S Townsend, S Shidfar, A South.
PHPT, Thailand: TR Cressey, S Chalermpantmetagul, R Peongjakta, K Than-in-at, S Chailert, K Seubmongkolchai, S Thammajitsagul, W Sripaoraya (L), C Kasemrat (L), A Upra (P), G Jourdain, M Lallemant, N Ngo-Giang-Huong, S Le Coeur.
Immunology and Virology Advisory Group: A De Rossi, N Klein, M.A.Muñoz Fernandez, E Nastouli, N Ngo, D Pillay.
Qualitative Substudy Group: S Bernays, S Paparini, T Rhodes, J Seeley.
Endpoint Review Committee: K Butler, DM Gibb, V Musiime.
Communications: Magda Conway, CHIVA (Children's HIV Association).
Recruiting Sites:
Argentina: Hospital Dr JP Garrahan / Fundación Helios Salud, Buenos Aires: R Bologna, J Da Bouza, D Mecikovsky, G Sotera, A Mangano, M Moragas.
Belgium: M Hainaut, E Van Der Kelen, S Vandenwijngaert.
Denmark: V Rosenfeldt, N Valerius, L Jensen.
Germany: J W Goethe University Frankfurt: C Koenigs, S Schultze-Strasser, R Linde, K Mantzsch
Ireland: Our Lady's Children's Hospital, Dublin: K Butler, P Gavin, R Leahy, A Rochford, M Goode, A Walsh, E Hyland, M O'Connor.
Spain: I Garcia Mellado Hospital 12 de Octubre, Madrid: P Rojo, M I Gonzalez Tomé; Hospital Sant Joan De Déu, Barcelona: C Fortuny Guasch, A Noguera Julian, P Santin Riba, A.Murciano Cabeza; Hospital La Fe, Valencia: M D Perez Tamarit, M C Otero Reigada, F Castera Brugada, I Segarra Granell, R Amigo Moreno; Hospital La Paz, Madrid: M J Mellado Peña, M Garcia Lopez Hortelano, M I De José Gomez, L Escosa; Universitario de Getafe: S Guillen Martin, L M Prieto Tato; Hospital Clínico San Carlos, Madrid: JT Ramos; Biobanco Gregorio Marañon, Madrid: M A Muñoz Fernandez, J L Jimenez Fuentes, C Gómez Rico, A Garcia Torre.
Thailand: The HIV Netherlands Australia Thailand Research Collaboration (HIV-NAT): T Bunupuradah, J Ananworanich, T Pitimahajanaka, N Thammajaruk; (PHPT): Kalasin Hospital: S Srirojana, D Dornngern, N Kunchanarong, L Mongkun; Regional Health Promotion Center Region 6, Khon Kaen: S Hanpinitsak, K Aue-apisak,P Kangsavon, M Shevasateanchai.
UK: Great Ormond Street Hospital, London: N Klein, J Kenny, A Turkova, D Shingadia, J Flynn, M Clapson, K Parkes, E Howley, L Spencer-Walsh; Evelina Children's Hospital, London: E Menson, R Cross, C Duncan, V Timms, E Reus, A Callaghan, S Tomlin, E Jones; Institute of Child Health, London: H Poulsom, N Klein, L Carter; Royal Infirmary Bristol: J Bernatoniene, A Finn, E Clarke, F Manyika, L Hutchison, H Smee, L Ball, K Stevenson; Heartlands Hospital Birmingham: S Welch, S Hackett, G Gilleran, J Daglish, L Horton, K Gandhi; Queen's Medical Centre Nottingham: A Smyth, J Smith, A Short, L Fear, S Stafford, S Hodgson, Y Taha; Leicester Royal Infirmary, S Bandi, J Philps, J Bwire, J Gardener; St. George's Hospital London: K Doerholt, K Prime, M Sharland, S Donaghy, L Spencer-Walsh, S Storey, O Okolo, D Rolfe; London School of Hygiene and Tropical Medicine: S

Bernays, T Rhodes, J Seeley. University College London Hospital (Department of Virology): E Nastouli, S Kirk, P Grant, B Ferns, J Garson.
Uganda: Joint Clinical Research Centre Kampala: C Kityo, V Musiime, H Kizito, A Nanduudu, A Drasiku, E Kaudha, S Senyonjo, I Obella, M Odera, P Oronon, H Nakyambadde, P Kyobutungi, O Senfuma, D Eram, J Nkalubo, L Nakiire, M Nabalaama, I Ssewanyana, G Pimundu, P Segonga, B Nakalawa, L Mugarura, A Kwaga, J Kasozi, M Ojok, J Namusanje; MRC/UVRI Uganda Research Unit on AIDS Entebbe: M Ndagire, S Namukwaya.
Ukraine: City AIDS Center Kiev: A Volokha, I Raus, O Mostovenko, N Chentsova. **USA:** St. Jude's Children's Research Hospital Memphis: P Flynn, R Dallas, T Wride, J Utech, S Ost, A Gaur, K Knapp, N Patel, M Shenep, T Culley, M Griffith, S Carr, C Longserre.

Ultrasensitive quantitative virological assays: additional information on methods

The ultrasensitive quantitative in-house HIV-1 RNA assay used the Qiagen QIASymphonySP for automated extraction of HIV-1 RNA from 1000µl of plasma sample using the cellfree1000 protocol and the QIAsymphony DSP Virus/Pathogen Midi Kit. The elution volume was set to 60µl, and the assay used targets for HIV-1 LTR and HIV-1 POL as well as brome mosaic virus (BMV) RNA as an internal control, which was introduced extraction stage at 6pg per sample.

The RT-PCR reaction was set up as a 50µl volume using the Invitrogen Superscript III one step RT-PCR kit containing 20µl of the extracted RNA and 0.2µM of the following primers and probes:
HIV1LTRTaq1 - 5' GCCTCAATAAAGCTTGCCTTGA;
HIV1LTRTaq2 - 5' GGCGCCACTGCTAGAGATTTT;
HIV1LTRProbe - 5' FAM TGTGACTCTGGTAACTAGAGATCCCTCAGAC TAMRA;
HIV1POLTaq1 - 5' TGTACCAGTAAAATTAAAGCCAGGAA;
HIV1POLTaq2 - 5' TATGGATTTTCAGGCCCAATT;
HIV1POLProbe - 5' FAM- TGGATGGBCCAARRGTYAAACARTGGCCATT –TAMRA;
BMVTaq1 - 5'-GTTCACCGATAGACCGCTG;
BMVTaq2 - 5'-AAGAGCCCGGAATGTCAA;
BMVProbe - 5'-*VIC*-CCTCAAGCTGAAATGGCACGGATG-*TAMRA*.
The RT-PCR reaction was cycled in an Applied Biosystems Prism 7500 with the following cycling conditions: 50°C for 15 minutes; 95°C for 2 minutes, followed by 45 cycles of 95°C for 15 seconds 60°C for 40 seconds. Data collection of FAM and VIC signals was set at the 60°C step. The quantification was based on an in house standard curve calibrated against the WHO HIV International standard, but expressed as copies/ml, using a conversion factor of 0.56 HIV-1 copies/IU.

The quantitative total HIV-1 DNA assay was performed as described elsewhere.[1] The assay has a lower limit of detection of approximately 50 copies/million cells.

Reference:

1. Smith NM, Mlcochova P, Watters SA, et al. Proof-of-principle for immune control of global HIV-1 reactivation in vivo. Clin Infect Dis 2015;61:120-8.

Supplementary Table 1: **Centres participating in the BREATHER trial**

**Centre name**	**Country**	**Number of participants enrolled**	**Total number of participants enrolled from country**
Fundacion Helios Salud, Buenos Aires<br>Hospital Dr. J.P. Garrahan, Buenos Aires	Argentina<br>Argentina	7<br>4	11
St. Pierre University Hospital, Brussels	Belgium	2	2
Hvidovre Hospital	Denmark	3	3
J W Goethe University Frankfurt	Germany	3	3
Our Lady's Children's Hospital, Dublin	Ireland	3	3
12 de Octubre, Madrid<br>Carlos III, Madrid<br>Universitario de Getafe<br>Hospital La Fe, Valencia<br>Sant Joan de Déu, Barcelona	Spain<br>Spain<br>Spain<br>Spain<br>Spain	5<br>2<br>2<br>1<br>1	11
HIV-NAT, Bangkok<br>Kalasin hospital<br>Khon Kaen	Thailand<br>Thailand<br>Thailand	25<br>7<br>4	36
Joint Clinical Research Centre (JCRC), Kampala	Uganda	70	70
Kiev City AIDS Centre	Ukraine	20	20
Great Ormond Street Hospital, London<br>Heartlands Hospital, Birmingham<br>St. George's Hospital, London<br>St. Thomas' Hospital, London<br>Bristol Royal Hospital for Children<br>Leicester University Hospital<br>Queen's Medical Centre, Nottingham	United Kingdom<br>United Kingdom<br>United Kingdom<br>United Kingdom<br>United Kingdom<br>United Kingdom<br>United Kingdom	10<br>4<br>3<br>3<br>2<br>2<br>2	26
St. Jude Children's Research Hospital, Memphis	United States of America	14	14

Supplementary Table 2: **Mean changes in MCV (fL), platelets ($x10^9/L$), and LDL cholesterol (mg/dL) from randomisation to week 48 assessment by use of zidovudine at baseline**

A: **Mean change in MCV (fL) from randomisation to week 48 assessment by use of zidovudine at baseline**

	week	SCT			CT			p value
		n	mean	se	n	mean	se	
On ZDV	12	51	-4.7	1.0	53	-0.4	1.0	0.0029
	24	52	-4.0	0.6	54	-0.4	0.5	<0.0001
	36	51	-3.4	0.6	53	-0.7	0.6	0.0013
	48	51	-4.4	0.6	52	-1.2	0.6	0.0004
Off ZDV	12	41	-1.9	0.6	45	-1.7	0.6	0.7926
	24	43	-3.0	0.7	43	-1.5	0.7	0.1500
	36	43	-3.0	0.8	41	-2.2	0.8	0.4485
	48	43	-2.7	0.8	41	-2.3	0.8	0.7201

Change calculated using linear regression, adjusting for baseline value, presenting mean change from baseline. CT=continuous therapy. SCT=short cycle therapy. n=number of patients. se=standard error

B: **Mean change in number of platelets ($x10^9/L$) from randomisation to week 48 assessment by use of zidovudine at baseline**

	week	SCT			CT			p value
		n	mean	se	n	mean	se	
On ZDV	12	51	-7.2	9.5	53	15.1	9.4	0.0991
	24	51	-7.7	7.8	54	3.0	7.5	0.3238
	36	52	-12.9	6.3	53	-12.3	6.2	0.9465
	48	51	-8.5	8.9	52	-3.2	8.8	0.6762
Not on ZDV	12	44	-14.4	6.7	46	10.9	6.5	0.0079
	24	46	-19.9	6.6	45	6.3	6.7	0.0067
	36	46	-21.1	9.0	43	13.7	9.3	0.0087
	48	44	-19.9	9.5	42	20.8	9.7	0.0036

Change calculated using linear regression, adjusting for baseline value, presenting mean change from baseline. CT=continuous therapy. SCT=short cycle therapy. n=number of patients. se=standard error

C: **Mean change in LDL cholesterol (mg/dL) from randomisation to week 48 assessment**

week	SCT			CT			p value
	n	mean	se	n	mean	se	
12	20	2.9	8.0	18	8.2	8.2	0.6462
24	81	4.3	1.8	83	-3.8	1.8	0.0014
36	16	-0.9	4.0	16	-3.0	3.9	0.6982
48	89	1.3	1.7	92	-0.3	1.6	0.4948

Change calculated using linear regression, adjusting for baseline value, presenting mean change from baseline. CT=continuous therapy. SCT=short cycle therapy. n=number of patients. se=standard error

Supplementary Table 3: **Results from biomarkers substudy**

Marker	Week 0				Week 48				SCT vs CT at week 48§ [95% CI]	p value§
	SCT		CT		SCT		CT			
	N	Median (IQR)	N	Median (IQR)	N	Median (IQR)	N	Median (IQR)		
CRP*	95	631.2 (303.8, 2676.1)	99	621.6 (260.8, 2164.1)	95	691.1 (309.0, 2530.7)	94	678.2 (309.7, 3015.1)	-0.3 [-0.7, 0.2]	0.3
SAA*	95	823.9 (379.8, 2248.5)	99	678.0 (427.6, 1701.8)	95	701.1 (371.0, 2416.6)	94	754.8 (359.6, 2407.6)	-0.1 [-0.6, 0.4]	0.7
sICAM-1*	95	396.4 (320.7, 565.5)	99	421.1 (360.6, 514.2)	95	418.3 (318.0, 615.5)	94	426.3 (330.0, 518.8)	0.0 [-0.1, 0.2]	0.6
sVCAM-1*	95	486.0 (398.1, 618.8)	99	486.5 (405.1, 614.4)	95	468.7 (480.1, 391.3)	94	480.1 (399.2, 632.5)	0.0 [-0.1, 0.2]	0.7
IL-10*	95	0.7 (0.5, 1.0)	99	0.8 (0.6, 1.1)	94	0.7 (0.6, 1.1)	94	0.8 (0.6, 1.2)	-0.1 [-0.3, 0.1]	0.4
IL-6*	95	0.6 (0.4, 0.9)	99	0.6 (0.4, 0.9)	93	0.6 (0.4, 1.0)	94	0.6 (0.4, 1.0)	-0.1 [-0.3, 0.2]	0.6
IL-8*	95	3.3 (2.1, 5.9)	99	4.1 (2.4, 7.9)	94	4.1 (2.0, 6.1)	94	4.6 (2.8, 8.8)	-0.2 [-0.5, 0.1]	0.2
MCP-1*	95	81.1 (57.0, 108.7)	99	81.8 (56.5, 112.8)	94	82.3 (64.6, 110.8)	94	91.3 (66.7, 116.6)	-0.1 [-0.2, 0.0]	0.1
TNF_a*	95	3.1 (2.3, 3.7)	99	2.9 (2.4, 3.7)	94	3.0 (2.0, 3.6)	94	3.1 (2.5, 4.0)	-0.1 [-0.2, 0.0]	0.1
VEGF*	95	39.3 (25.7, 69.1)	99	37.3 (24.7, 68.2)	94	41.4 (28.1, 61.7)	94	43.9 (29.1, 100.7)	-0.2 [-0.4, 0.1]	0.2
IL-1Ra*	95	233.8 (179.2, 368.2)	99	249.4 (179.6, 382.3)	95	265.0 (183.0, 372.0)	94	259.9 (186.0, 396.7)	0.0 [-0.2, 0.2]	1.0
E-SEL#	95	7.8 (5.8, 11.7)	99	7.0 (5.4, 10.6)	95	7.5 (5.7, 11.7)	94	7.6 (5.5, 10.9)	-0.0 [-0.1, 0.1]	0.5
P-SEL#	95	50.8 (35.6, 78.2)	99	49.2 (33.5, 85.6)	95	55.0 (35.4, 83.5)	94	49.4 (36.1, 89.3)	-0.0 [-0.2, 0.1]	0.7
ICAM-3#	95	1.1 (0.8, 1.4)	99	1.0 (0.8, 1.3)	95	1.2 (0.9, 1.6)	94	1.1 (0.8, 1.4)	0.1 [-0.1, 0.2]	0.3
TM#	95	3.1 (2.5, 4.0)	99	3.0 (2.3, 3.7)	95	3.2 (2.5, 4.2)	94	3.2 (2.4, 4.0)	0.0 [-0.1, 0.1]	0.4
Ang-1*	95	5708 (2312, 12717)	99	5274 (2334, 11774)	95	4366 (2121, 11533)	94	5146 (2098, 11303)	-0.1 [-0.4, 0.2]	0.7
Ang-2*	95	5401 (4103, 7061)	99	5292 (4234, 7006)	95	5902 (4409, 7672)	94	5610 (4244, 6983)	0.0 [-0.1, 0.1]	0.4
D-dimer#	93	69.1 (3.13, 135.4)	97	65.7 (4.82, 180.3)	92	46.2 (2.0, 126.7)	92	66.4 (3.1, 197.2)	-0.5 [-1.0, -0.0]	0.05
TF*	94	38.7 (33.2, 44.3)	99	40.6 (32.3, 47.3)	95	42.8 (36.4, 48.3)	93	40.8 (34.0, 48.8)	0.0 [-0.0, 0.1]	0.5

Ang-1=angiopoietin-1.Ang-2=angiopoietin-2. CRP=C-reactive protein. E-SEL=E-selectin. ICAM-3=intercellular adhesion molecule 3.IL-1Ra=interleukin-1 receptor antagonist. IL-6=Interleukin-6. IL-8= interleukin-8. IL-10=interleukin-10. MCP-1=monocyte chemoattractant protein-1.P-SEL=P-selectin. SAA=serum amyloid A. TM=trombomodulin. TF=tissue factor. TNF- α=tumour necrosis factor-α. sVCAM-1=soluble vascular cell adhesion molecule-1. VEGF= vascular endothelial growth factors. §From linear regression models of log(outcome) at week 48, adjusting for baseline log(outcome). *measured in pg/ml. #measured in ng/ml.

Supplementary Table 4: **Comparison of adherence difficulties between baseline and end of study in short-cycle therapy group.**

**Reasons for difficulties taking medications***	**Baseline N=76$**	**End of study N=76$**	**p value#**
Remembering to take meds	21	16	0·42
Timing of meds	18	14	0·50
Number of tablets	8	5	0·55
Size of tablets	9	5	0·39
Difficulty swallowing	9	3	0·15
Amount of syrup	1	1	1·00
Taste of meds	12	7	0·23
Side-effects	10	5	0·30
Different routine (weekends)	8	2	0·11
Different routine (week days)	4	2	0·69
School/college days	5	1	0·13
School/college holidays	6	4	0·75
Staying with friends/family	12	17	0·33
Going out with friends	15	2	0·001
Other	11	5	0·21

Data are number of participants. *From acceptability questionnaires filled in by children and adolescents. $76 participants completed questionnaires at both time points. #McNemar's test
